# Telomere-to-telomere genome assembly and multiomics analyses illustrate the high accumulation of quercetin glucosides in tetraploid *Descurainia sophia*

**DOI:** 10.1093/hr/uhaf335

**Published:** 2025-12-03

**Authors:** Weifeng Wu, Jianyong Wang, Chengcheng Cai, Xiaoyu Song, Hua Li, Tao Zhang, Meixin Xiong, Ying Wang, Jie Zhang, Bingbing Li, Lei Zhang, Feng Li, Mingkun Huang, Wei Li, Feng Cheng, Danyu Kong, Yi Liu

**Affiliations:** Jiangxi Provincial Key Laboratory of Plant Germplasm Resources Innovation and Genetic Improvement, Lushan Botanical Garden, Jiangxi Province and Chinese Academy of Sciences, Jiujiang 332900, Jiangxi, China; Lushan Botanical Garden, Jiangxi Province and Chinese Academy of Sciences, Jiujiang 332900, Jiangxi, China; Lushan Botanical Garden, Jiangxi Province and Chinese Academy of Sciences, Jiujiang 332900, Jiangxi, China; Jiangxi Key Laboratory for Sustainable Utilization of Chinese Materia Medica Resources, Lushan Botanical Garden, Jiangxi Province and Chinese Academy of Sciences, Jiujiang 332900, Jiangxi, China; State Key Laboratory of Vegetable Biobreeding, Key Laboratory of Biology and Genetic Improvement of Horticultural Crops of the Ministry of Agriculture and Rural Affairs, Institute of Vegetables and Flowers, Chinese Academy of Agricultural Sciences, 12 Zhongguancun South Street, 100081 Beijing, China; Shenzhen Branch, Guangdong Laboratory of Lingnan Modern Agriculture, Key Laboratory of Synthetic Biology, Ministry of Agriculture and Rural Affairs, Agricultural Genomics Institute at Shenzhen, Chinese Academy of Agricultural Sciences, 518120 Shenzhen, China; Lushan Botanical Garden, Jiangxi Province and Chinese Academy of Sciences, Jiujiang 332900, Jiangxi, China; Jiangxi Key Laboratory for Sustainable Utilization of Chinese Materia Medica Resources, Lushan Botanical Garden, Jiangxi Province and Chinese Academy of Sciences, Jiujiang 332900, Jiangxi, China; Lushan Botanical Garden, Jiangxi Province and Chinese Academy of Sciences, Jiujiang 332900, Jiangxi, China; Jiangxi Key Laboratory for Sustainable Utilization of Chinese Materia Medica Resources, Lushan Botanical Garden, Jiangxi Province and Chinese Academy of Sciences, Jiujiang 332900, Jiangxi, China; Lushan Botanical Garden, Jiangxi Province and Chinese Academy of Sciences, Jiujiang 332900, Jiangxi, China; Jiangxi Key Laboratory for Sustainable Utilization of Chinese Materia Medica Resources, Lushan Botanical Garden, Jiangxi Province and Chinese Academy of Sciences, Jiujiang 332900, Jiangxi, China; Institute of Biotechnology and Nuclear Technology Research, Sichuan Academy of Agricultural Sciences, Chengdu 610061, Sichuan, China; Institute of Biotechnology and Nuclear Technology Research, Sichuan Academy of Agricultural Sciences, Chengdu 610061, Sichuan, China; Bellagen Biotechnology Co. Ltd., Jinan 250307, Shandong, China; State Key Laboratory of Vegetable Biobreeding, Key Laboratory of Biology and Genetic Improvement of Horticultural Crops of the Ministry of Agriculture and Rural Affairs, Institute of Vegetables and Flowers, Chinese Academy of Agricultural Sciences, 12 Zhongguancun South Street, 100081 Beijing, China; Bellagen Biotechnology Co. Ltd., Jinan 250307, Shandong, China; Lushan Botanical Garden, Jiangxi Province and Chinese Academy of Sciences, Jiujiang 332900, Jiangxi, China; Shenzhen Branch, Guangdong Laboratory of Lingnan Modern Agriculture, Key Laboratory of Synthetic Biology, Ministry of Agriculture and Rural Affairs, Agricultural Genomics Institute at Shenzhen, Chinese Academy of Agricultural Sciences, 518120 Shenzhen, China; State Key Laboratory of Vegetable Biobreeding, Key Laboratory of Biology and Genetic Improvement of Horticultural Crops of the Ministry of Agriculture and Rural Affairs, Institute of Vegetables and Flowers, Chinese Academy of Agricultural Sciences, 12 Zhongguancun South Street, 100081 Beijing, China; Jiangxi Provincial Key Laboratory of Plant Germplasm Resources Innovation and Genetic Improvement, Lushan Botanical Garden, Jiangxi Province and Chinese Academy of Sciences, Jiujiang 332900, Jiangxi, China; Lushan Botanical Garden, Jiangxi Province and Chinese Academy of Sciences, Jiujiang 332900, Jiangxi, China; Lushan Botanical Garden, Jiangxi Province and Chinese Academy of Sciences, Jiujiang 332900, Jiangxi, China; Jiangxi Key Laboratory for Sustainable Utilization of Chinese Materia Medica Resources, Lushan Botanical Garden, Jiangxi Province and Chinese Academy of Sciences, Jiujiang 332900, Jiangxi, China

## Abstract

Quercetin glucosides are important phytopharmaceutical metabolites in *Descurainia sophia* seeds, which are widely used in traditional herbal medicine. However, the key genes involved in quercetin glucoside biosynthesis in *D. sophia* have not been characterized. Herein, we present the telomere-to-telomere genomes of a tetraploid *D. sophia*, which accumulates high levels of quercetin glucoside, and a diploid *D. sophia*, which accumulates only trace amounts. Multiomics analyses and uridine diphosphate glucosyltransferase (UGT) enzyme assays revealed that the gene duplication and functional evolution of *Dscd6AG01520*, an UGT gene, led to high quercetin-3-*O*-β-d-glucoside and quercetin-3,7-*O*-β-d-diglucoside accumulation in tetraploid *D. sophia* seeds. Further UGT enzyme assays with the point mutations of Dscd6AG01520 showed that S213 was a critical amino acid for the enzymatic activity of Dscd6AG01520. In addition, we found that diploid *D. sophia* evolved from an ancestral crucifer karyotype through chromosome fusion and rearrangement. Collectively, our findings illuminate the mechanism of high quercetin glucoside accumulation in tetraploid *D. sophia*, clarify the origin of the diploid *D. sophia* genome, and provide valuable genomic resources for comparative genomics and research into polyploid evolution.

## Introduction

*Descurainia sophia* (L.) Webb ex Prantl, commonly known as flixweed, is an annual, self-compatible, dicotyledonous plant in the Brassicaceae family. *D. sophia* is native to Eurasia and is distributed from Portugal to China and in northern Africa [1]. The seeds of *D. sophia* are used in popular herbal remedies and are suitable for many food and industrial applications [2–5]. *D. sophia* seeds have medicinal benefits, acting as a laxative, febrifuge, expectorant, demulcent, and diuretic [2, 3, 5]. They also support heart health and recovery, helping treat asthma, fevers, bronchitis, edema, and dysentery [2, 3, 5]. As noted in the 2020 edition of the Chinese Pharmacopoeia, mature *D. sophia* seeds are utilized as a traditional Chinese medicine. In Iran, *D. sophia* seeds are traditionally used to produce a sweet drink that detoxifies the liver [6].

Flavonoids are a major class of secondary metabolites, possessing important pharmacological activities [7–11]. The flavonoids quercetin and quercetin glucosides, including isoquercetin (quercetin-3-*O*-β-d-glucoside, Q3G), quercimeritrin (quercetin-7-*O*-β-d-glucoside, Q7G), and quercetin 3,7-*O*-β-d-diglucoside (Q3,7G), are considered important phytopharmaceutical metabolites in *D. sophia* seeds. Quercetin, which cannot be produced in the human body, exhibits broad pharmacological effects, including anti-inflammatory, antioxidant, cardioprotective, and metabolic regulatory properties, along with anticancer, antiviral, and antiasthmatic activities [12–14]. However, the bioavailability of quercetin is relatively low because of its poor water solubility, chemical stability, and absorption profile [14, 15]. Glycosylation, which refers to the attachment of specific sugar moieties to secondary metabolites catalyzed by uridine diphosphate-dependent glycosyltransferases (UGTs), can enhance the water solubility of hydrophobic metabolites and significantly improve their bioavailability [16]. Q3G is a major glycosidic form of quercetin that exhibits significant pharmacological activity against cancer, oxidative stress, cardiovascular disorders, diabetes, and allergic reactions, with higher bioavailability than quercetin [10, 17–20]. Q7G is a natural quercetin glucoside that possesses *in vitro* anti-inflammatory, antioxidant, and anti-viral activities [10, 21, 22]. Another natural quercetin glucoside, Q3,7G, displays strong antioxidant and antileukemia activities [8, 9]. Although Q3G, Q7G, and Q3,7G have many important pharmacological activities, the UGTs involved in quercetin glucoside biosynthesis have not been characterized in *D. sophia*.

In the present study, metabolomics and metabolite content analysis demonstrated that tetraploid *D. sophia* seeds accumulated significantly higher levels of Q3G, Q7G, and Q3,7G than diploid *D. sophia* seeds, which contained only trace amounts. In contrast, the total quercetin content was comparable between diploid and tetraploid *D. sophia*. To identify the UGTs contributing to the high accumulation of Q3G and Q3,7G in tetraploid *D. sophia*, we assembled high-quality reference genomes for diploid and tetraploid *D. sophia*. *UGT* gene annotation, expression correlation analysis, and *in vitro* enzyme assays showed that the gene duplication and evolution of *Dscd6AG01520* contributed to high quercetin glucoside accumulation (Q3G and Q3,7G) in tetraploid *D. sophia*.

## Results

### Metabolite profile reveals high levels of quercetin glucosides in tetraploid *D. sophia*

**Figure 1:**
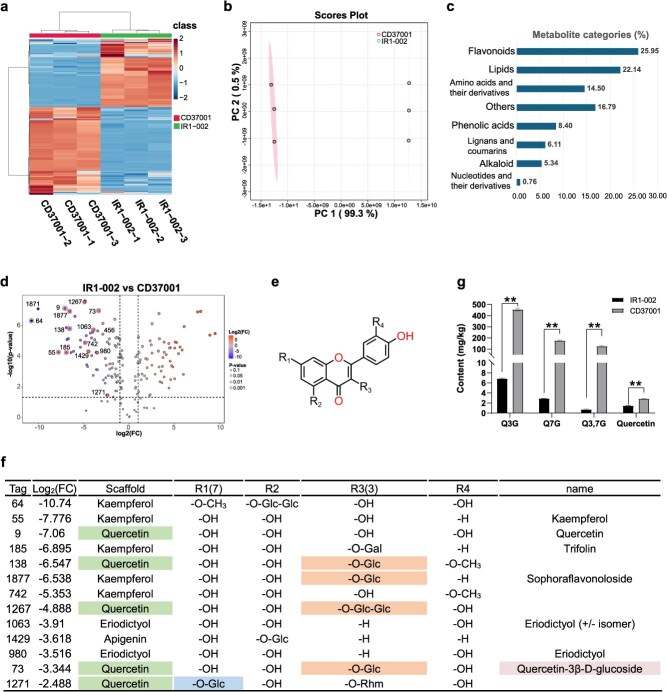
Metabolome analysis of two *D. sophia* cultivars. (a) Heatmap showing the differentially accumulated metabolites between IR1-002 and CD37001 seeds. (b) Principal component analysis of metabolites in two cultivars. (c) Percentage and type of differentially accumulated metabolites annotated from the metabolome. (d) Volcano plot showing the metabolites with higher accumulation in CD37001 than in IR1-002. (e) Scaffold structure of flavonoids. (f) Information on glycosylated flavonoids with higher accumulation in CD37001. (g) Quercetin and quercetin glucoside contents in CD37001 and IR1-002. Data are means ± standard deviation (SD) (*n* ≥ 3); asterisks indicate statistical significance according to the results of a *t*-test, where ^*^P < 0.05, ^**^P < 0.01.

Our laboratory collected two *D. sophia* germplasm resources, of which one (CD37001) was from China and the other (IR1-002) was from Iran. Karyotype analysis revealed that IR1-002 was diploid and that CD37001 was tetraploid (Supplementary Fig. S1). To study the metabolite profile of the *D. sophia* seeds, we conducted untargeted liquid chromatography–mass spectrometry (LC–MS) metabolomic analysis to compare the nonvolatile metabolites between diploid (IR1-002) and tetraploid *D. sophia* (CD37001) seeds. A total of 2666 metabolic signals were identified (Fig. 1a), and principal component analysis revealed distinct metabolic profiles between the two *D. sophia* types (Fig. 1b). Of the 131 differentially accumulated metabolites, 34 were flavonoids, and lipids (29), amino acids and their derivatives (19), phenolic acids (11), lignans and coumarins (8), alkaloids (7), nucleotides and their derivatives (1), and others (22) were also identified (Fig. 1c). Among these metabolites, the content of 51 differentially accumulated metabolites in IR1-002 was less than one-fourth of that in CD37001 (Fig. 1d). The 13 flavonoids with low accumulation in IR1-002 displayed a notable trend in which many had a quercetin scaffold (5/13) and glycosylation at position 3 (R3, 4/13) (Fig. 1e and f). Therefore, a quercetin scaffold and 3-glycosylation were the main features of metabolites with higher accumulation in CD37001 than in IR1-002. We also observed that the 7-glycosylated quercetin content was higher in CD37001 than that in IR1-002 (Fig. 1e and f).

To further measure the absolute content of quercetin glucoside and confirm the difference in quercetin glucoside contents between CD37001 and IR1-002, the contents of quercetin, Q3G, Q7G, and Q3,7G in CD37001 and IR1-002 seeds were quantified based on LC–MS analysis using the corresponding reference standards. In CD37001, the contents of quercetin, Q3G, Q7G, and Q3,7G were 2.83, 454.84, 176.02, and 125.96 mg/kg, respectively, whereas in IR1-002, they were 1.48, 6.88, 2.91, and 0.71 mg/kg, respectively (Fig. 1g). These results showed that the content of quercetin, which is the precursor of quercetin glucosides, was less than twice as high in CD37001 compared to IR1-002. The contents of quercetin glucosides, including Q3G, Q7G, and Q3,7G, were 66-, 60-, and 178-fold higher, respectively, in CD37001 than in IR1-002.

To further investigate the substantial disparity in quercetin glucosides (Q3G, Q7G, and Q3,7G) between diploid and tetraploid *D. sophia*, this study conducted genome sequencing and association analyses to elucidate the quercetin glucoside biosynthesis pathway in *D. sophia*.

### Chromosome-level genome assemblies of diploid and tetraploid *D. sophia*

**Figure 2:**
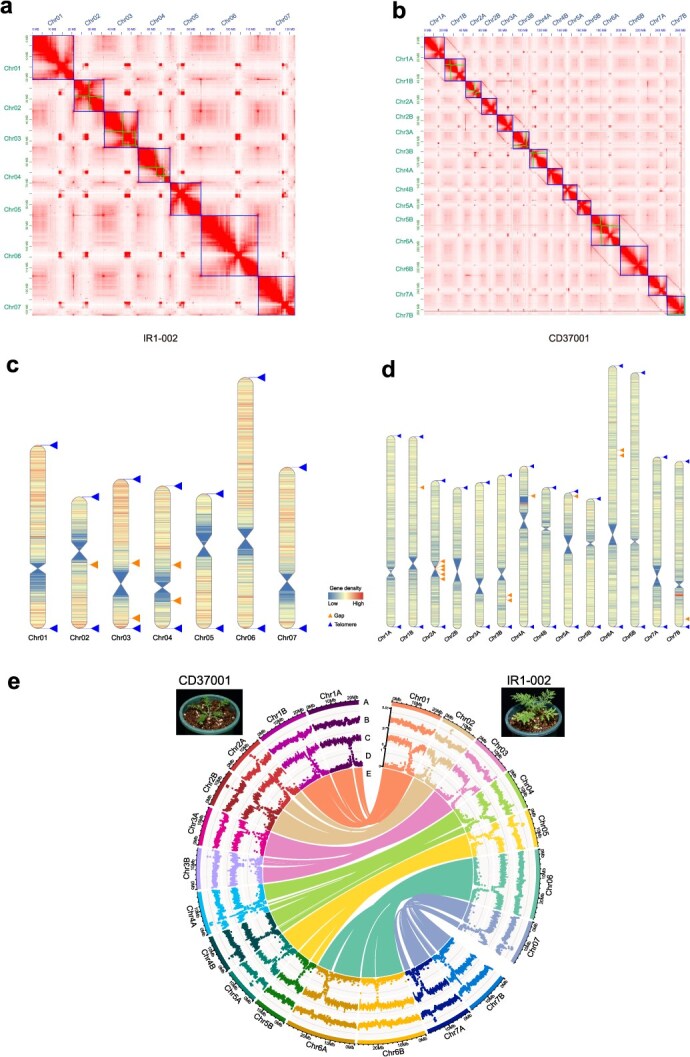
High-quality T2T genome assembly of two *D. sophia* cultivars. (a) Hi-C interactive heatmap of IR1-002 genome assembly. (b) Hi-C interactive heatmap of CD37001 genome assembly. (c) Telomere detection map of IR1-002. (d) Telomere detection map of CD37001. Blue and orange triangles represent telomeres and gaps within the assembled chromosomes, respectively; red indicates high gene density, while blue represents low gene density. (e) Genome information of IR1-002 and CD37001, including chromosome ideogram information (A), GC content (B), gene density (C), TE density (D), and collinear genomic blocks (E).

To systematically investigate the quercetin glucoside biosynthesis pathway, we assembled the genomes of IR1-002 and CD37001 using data generated from multiple sequencing platforms, including 28.59 Gb (213×) and 33.97 Gb (119×) PacBio HiFi long reads for IR1-002 and CD37001, respectively, and 130.32 Gb (970×) and 123.63 Gb (433×) Hi-C reads for IR1-002 and CD37001, respectively. The total lengths of the diploid and tetraploid *D. sophia* genome assemblies were 134.4 and 285.8 Mb, respectively, with contig N50 values of 18.57 and 18.53 Mb, respectively (Table 1). The Hi-C data were employed to anchor 98.88% of the assembled diploid *D. sophia* contigs to 7 pseudochromosomes, while 93.05% of the assembled tetraploid *D. sophia* contigs were anchored to 14 pseudochromosomes (Fig. 2a–e and Table 1). The strong signal along the diagonal of the interaction between proximal regions reflected the high quality of the Hi-C assemblies for both diploid and tetraploid *D. sophia* (Fig. 2a and b). The benchmarking universal single-copy ortholog (BUSCO) values were 99.0% and 99.4% for IR1-002 and CD37001, respectively, demonstrating the high completeness of the genomes.

**Table 1:** Assembly and annotation statistics of *D. sophia* genomes

	IR1-002	CD37001
Genome size (Mb)	134.4	285.8
Contig N50 (Mb)	18.57	15.61
Contig number	42	338
Assembly BUSCO (brassicales_odb10)	99.0%	99.4%
GC content	36.14%	36.06%
Repeat percentage	17.25%	16.39%
Predicted gene number	28 465	66 035
Annotation BUSCO (brassicales_odb10)	97.1%	97.0%

We applied the quarTeT toolkit to characterize and predict the telomeres and centromeres in the IR1-002 and CD37001 genomes. A total of 14 telomere regions (AAACCCT) and 7 centromeric regions were identified in the IR1-002 genome (Fig. 2c), whereas 27 telomere regions and 14 centromeric regions were identified in the CD37001 genome (Fig. 2d). In the CD37001 genome, one telomere was not detected on chromosome 2B. In addition, the IR1-002 genome exhibited five gaps in three pseudochromosomes (chromosomes 4, 5, and 7), while the CD37001 genome displayed 13 gaps in seven pseudochromosomes. Ultimately, we successfully assembled telomere-to-telomere (T2T) reference genomes for diploid *D. sophia* IR1-002 and tetraploid *D. sophia* CD37001 (Fig. 2c and d).

Transposable element (TE) annotation identified 23.19 and 46.84 Mb of repetitive elements, which occupied approximately 17.25% and 16.39% of the IR1-002 and CD37001 genomes, respectively (Table 1). We further annotated the gene model by combining *de novo* prediction, homology search, and RNA sequencing (RNA-seq) data alignment. The IR1-002 and CD37001 genomes were predicted to contain 28 465 and 66 035 protein-coding genes, respectively. BUSCO values of 97.1% and 97.0% were obtained for the IR1-002 and CD37001 gene sets, respectively, indicating the high quality of the gene annotations.

### *D. sophia* genome evolved from an ancestral crucifer karyotype through the fusion of two chromosomes

**Figure 3:**
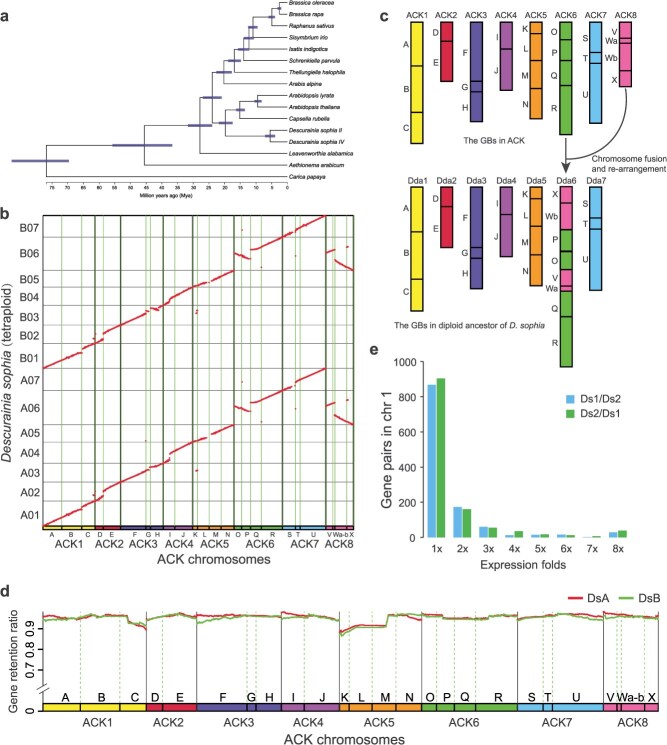
Genome evolution and subgenome dominance of *D. sophia*. (a) Phylogenetic relationships between *D. sophia* and other Brassicaceae species. (b) Syntenic fragments between tetraploid *D. sophia* and ACK chromosomes. (c) Deduced scenario in which the *D. sophia* chromosomes were derived from the ACK chromosomes. Ancestral chromosomes ACK6 and ACK8 fused and were subsequently rearranged to form the current chromosome 6. Dds: diploid ancestor of *D. sophia*. (d) Gene retention ratio in the two subgenomes (DsA and DsB) of tetraploid *D. sophia* compared to the ACK genome. (e) Number of dominantly expressed paralogs between the two subgenomes on chromosome 1. Blue denotes genes in DsA (A subgenome) that are dominantly expressed over their paralogs in DsB (B subgenome), while green indicates genes in DsB that are dominantly expressed over their paralogs in DsA.

Genome comparisons were performed between the 2 *D. sophia* genomes and 13 representative Brassicaceae genomes using the *Carica papaya* genome as an outgroup. We identified 104 syntenic gene families in all 16 genomes and 9097 synonymous nucleotide positions within the syntenic gene families. Using these synonymous loci, we constructed a phylogenetic tree that placed diploid and tetraploid *D. sophia* in Brassicaceae lineage I in a position close to *Capsella rubella* (Fig. 3a). *C. rubella* evolved from an ancestor with an ancestral crucifer karyotype (ACK) genome structure [23–25]. The ACK structure, featuring eight chromosomes, is believed to be the genome structure of the diploid ancestor of all Brassicaceae species [25]. These data suggest that the ancestral genome of *D. sophia* has a close evolutionary relationship with the ACK.

Genomic synteny analysis between the genomes of diploid/tetraploid *D. sophia* and *Arabidopsis thaliana* identified 20 545 and 39 597 syntenic gene pairs, respectively. The syntenic fragments between the genomes of *D. sophia* and *A. thaliana* were transferred to those between *D. sophia* and ACK based on the Brassicaceae genomic block (GB) system. The GB system was constructed in Brassicaceae to facilitate comparative genomic studies, with 22 GBs (A–X) defined in ACK using the genes and genomic fragments of *A. thaliana* as a reference [25]. To confirm the ACK origin of the diploid ancestor of *D. sophia*, we mapped GB information from the ACK genome to the *D. sophia* genome. For each of the 22 ancestral GBs in ACK, one and two copies were identified in diploid and tetraploid *D. sophia*, respectively (Fig. 3b and Supplementary Fig. S2). The 1:1 relationship between the genomes of diploid *D. sophia* and ACK indicated that no whole-genome duplication event occurred in diploid *D. sophia* after its divergence from ACK. Further comparison of GB associations between the genomes of *D. sophia* and ACK revealed that the vast majority (88.24%) of GB associations found in ACK were also detected in *D. sophia* (Fig. 3b). We revealed that six ancestral ACK chromosomes, namely ACK1, ACK2, ACK3, ACK4, ACK5, and ACK7, were inherited as the six chromosomes in the diploid ancestor of *D. sophia* (Fig. 3c), whereas the ancestral chromosomes ACK6 and ACK8 were fused and rearranged to form a single chromosome in *D. sophia* (Fig. 3c). This demonstrated that the diploid ancestor of *D. sophia* evolved from the ACK genome through the merging and rearrangement of two ACK chromosomes. Consistently, when we further applied an updated ancestral genome reconstruction from ACK, tAKI [26], the results were in full agreement with the ACK-based inference (Supplementary Fig. S3a–c), confirming the robustness of this evolutionary scenario.

We further investigated whether the two subgenomes in tetraploid *D. sophia* displayed subgenome dominance. Using the ACK genome as a reference, we compared the ratios of retained genes between the two subgenomes of tetraploid *D. sophia* along each ACK chromosome. We observed a high ratio (>90%) of retained genes in both subgenomes (Fig. 3d), which implied that the two subgenomes did not undergo extensive gene fractionation following tetraploidization. No significant differences were observed in the retained gene ratio between the two subgenomes, indicating the absence of subgenome dominance in tetraploid *D. sophia*. The near-equal gene retention ratio between the two subgenomes may be attributed to a relatively recent tetraploidization event. This was supported by the major peak in the synonymous substitution rate (*Ks*) distribution of 19 531 paralogs in tetraploid *D. sophia* at 0.12 (Supplementary Fig. S3d). We further compared the gene expression levels in siliques between syntenic paralogs in the two subgenomes and did not find a pattern in which there were significantly more genes on one subgenome that were more highly expressed than their homoeologs in the other subgenome (Fig. 3e). Similar results were consistent across other tested tissues, including roots, stems, leaves, flowers, and seeds (Supplementary Fig. S4). These results suggested that the two subgenomes of tetraploid *D. sophia* did not experience extensive gene fractionation or subgenome dominance. Furthermore, we compared the Ks distribution of *D. sophia* with those of the diploid *A. thaliana* and the autopolyploid *A. arenosa* (Supplementary Fig. S3d). We observed that the Ks peak (Ks = 0.12) corresponding to the polyploidization event in *D. sophia*, which was close to that of *A. arenosa* (Ks = 0.05), supporting the interpretation that this event represents a relatively recent autopolyploidization. In addition, we performed genomic synteny analysis between the diploid and tetraploid *D. sophia*. The result revealed a clear 1:2 correspondence between diploid and tetraploid chromosomes (Supplementary Fig. S5). Together, these data indicated that this was an autotetraploidization event.

### Duplication and evolution of *UGT*s lead to the high level of quercetin glucoside in tetraploid *D. sophia*

The similar quercetin contents and the substantial disparity in quercetin glucoside contents between diploid and tetraploid *D. sophia* indicate that UGTs with the function of catalyzing glycosylation contribute to the high quercetin glucoside accumulation in tetraploid *D. sophia*. To characterize the causal UGTs in IR1-002 and CD37001, siliques were collected at three developmental stages to measure the Q3G, Q7G, and Q3,7G contents and to perform RNA-seq analysis (Supplementary Fig. S6a). Based on the RNA-seq data and genome annotation, 117 and 216 *UGT* gene family members were identified in IR1-002 and CD37001, respectively. This study selected a total of 32 *UGT*s as candidate genes involved in quercetin glucoside biosynthesis for subsequent functional validation based on the correlation coefficients between the *UGT* expression level and the quercetin glucoside content (Supplementary Fig. S6b and Supplementary Tables S1, S2, and  S3).

**Figure 4:**
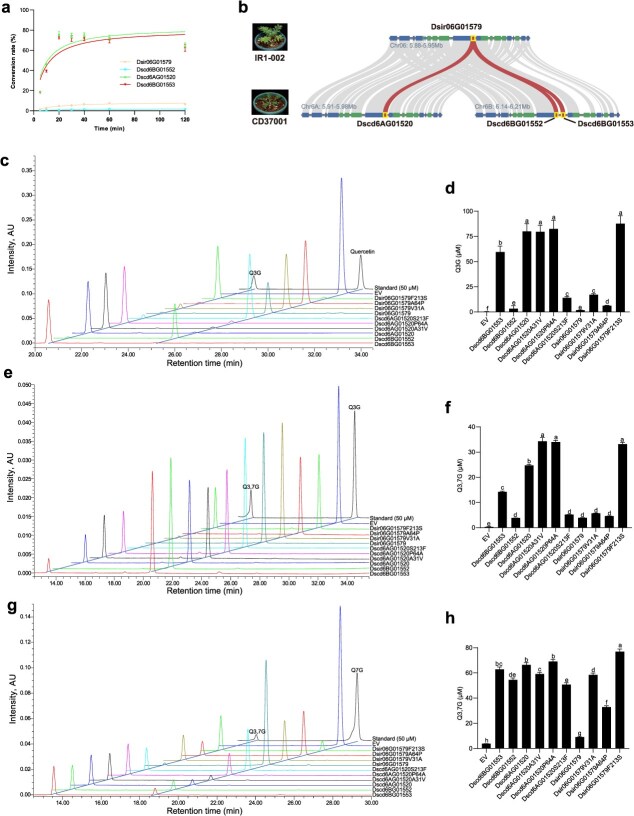
Enzymatic characterization of DsUGTs according to *in vitro* enzyme assays. (a) Dynamics of product formation in an *in vitro* enzymatic assay using quercetin (0.2 mM) as the substrate and UDP-glucose (0.5 mM) as the sugar donor. (b) Collinear relationships of Dsir06G01579 and its homoeologs in diploid (IR1-002) and tetraploid (CD37001) *D. sophia*. Syntenic blocks are connected by gray lines, and syntenic target genes are connected by red lines. (c) Overlay of HPLC chromatograms of the enzyme assay products of DsUGTs and their mutations toward quercetin. (d) Histogram of the content of the corresponding target product using quercetin as the substrate. (e) Overlay of HPLC chromatograms of the enzyme assay products of DsUGTs and their mutations toward Q3G. (f) Histogram of the content of the corresponding target product using Q3G as the substrate. (g) Overlay of HPLC chromatograms of the enzyme assay products of DsUGTs and their mutations toward Q7G. (h) Histogram of the content of the corresponding target product using Q7G as the substrate. Histograms present the statistical analysis of DsUGTs and their mutation enzyme activity. Data are means ± SD (*n* ≥ 3 technical repeats). Different lowercase letters in d, f, and h represent significant differences between samples within the same trait determined by one-way ANOVA with Tukey’s HSD test (*P* < 0.05). These experiments were repeated three times with similar results.

These 32 *UGT*s were expressed in *Escherichia coli*, and their enzyme activity was assessed using quercetin, Q3G, and Q7G as the substrates and UDP-glucose as the sugar donor. *In vitro* enzyme assays of recombinant UGTs demonstrated that only Dscd6BG01553 exerted enzymatic activity on quercetin, Q3G, and Q7G. Using authentic Q3G, Q7G, and Q3,7G as reference standards, the findings showed that Dscd6BG01553 catalyzed quercetin to form Q3G and Q3,7G, and catalyzed both Q3G and Q7G to form Q3,7G (Fig. 4a, c, e, and  g).

BLASTP analysis revealed that *Dscd6BG01553* had only one syntenic ortholog (*Dsir06G01579*) in IR1-002, whereas in CD37001, one homoeolog (*Dscd6AG01520*) and one tandem duplication (*Dscd6BG01552*) were identified (Fig. 4b). Among these four homoeologs, *Dscd6AG01520* shared the highest amino acid identity (99.33%) with *Dsir06G01579*. In contrast, *Dscd6BG01552* shared the lowest amino acid identity (85.33%) with *Dscd6BG01553* (Supplementary Table S4). Subsequently, Dsir06G01579, Dscd6AG01520, and Dscd6BG01552 were subjected to the same *in vitro* enzyme assays conducted for Dscd6BG01553. Using quercetin, Q3G, and Q7G as substrates, the results showed that Dsir06G01579, Dscd6AG01520, and Dscd6BG01552 exhibited enzyme activities (Fig. 4a, c–g, and Supplementary Table S5). Dscd6AG01520 showed the highest conversion rate with quercetin, Q3G, and Q7G as substrates to produce Q3G, Q3,7G, and Q3,7G, respectively, followed by Dscd6BG01553, Dscd6BG01552, and Dsir06G01579 (Fig. 4a, d, f, h, and Supplementary Table S5). Dsir06G01579 and Dscd6BG01552 displayed low and comparable conversion rates when utilizing quercetin and Q3G as substrates, while Dscd6BG01552 exhibited a much higher conversion rate than Dsir06G01579 using Q7G as the substrate (Fig. 4a, c, e, g, and Supplementary Table S5). Overall, the results indicate that Dscd6BG01553 and its homoeolog Dscd6AG01520 catalyze quercetin glucoside biosynthesis (Q3G and Q3,7G) with high efficiency.

**Figure 5:**
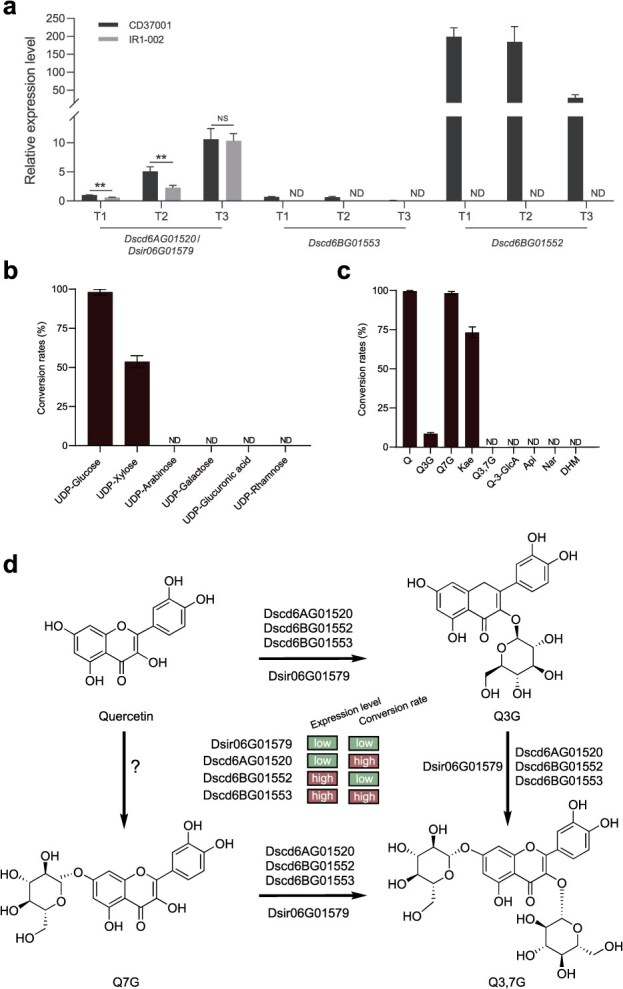
Role of *Dscd6AG01520* and its homoeologs in quercetin glucoside biosynthesis. (a) Expression patterns of *Dscd6AG01520* and its homoeologs in the siliques of diploid (IR1-002) and tetraploid (CD37001) *D. sophia* determined by quantitative RT-qPCR. Means ± SD; *n* = 3 technical repeats. These experiments were repeated three times with similar results. T1, T1 developmental stage; T2, T2 developmental stage; T3, T3 developmental stage. ^**^ represents significant differences (*P* < 0.01), and NS represents no significant difference between samples determined by a *t*-test (*P* < 0.05). (b) Sugar donor specificity of Dscd6AG01520. Conversion rates of glycosylation products using quercetin as the substrate. Values are presented as the means ± SD of three technical repeats. These experiments were repeated three times with similar results. (c) Substrate promiscuity of Dscd6AG01520. Conversion rates of glycosylated products toward nine substrates using UDP-glucose as the sugar donor. Values are presented as the means ± SD of three technical repeats. These experiments were repeated three times with similar results. Q, quercetin; Q3G, quercetin-3-*O*-β-d-glucoside; Q7G, quercetin-7-*O*-β-d-glucoside; Kae, kaempferol; Q3,7G, quercetin-3,7-*O*-β-d-diglucoside; Q-7-GlcA, quercetin-3-*O*-glucuronide; Api, apigenin; Nar, naringenin; DHM, dihydromyricetin. (d) Schematic of the biosynthetic pathway from quercetin to Q3,7G in *D. sophia*.

Quantitative reverse transcription polymerase chain reaction (RT-qPCR) was further conducted to determine the expression levels of these four *UGT* copies in the siliques, using *DsUBC21* as the internal reference [27]. *Dscd6AG01520*, *Dscd6BG01553*, and *Dsir06G01579* exhibited similar low expression levels in siliques at all three developmental stages. Conversely, the expression level of *Dscd6BG01552* was much higher (Fig. 5a). These results indicate that whole-genome and tandem duplication (*Dscd6BG01552*), functional evolution (*Dscd6AG01520* and *Dscd6BG01553*), and expression upregulation (*Dscd6BG01552*) contributed to the high Q3G and Q3,7G contents in tetraploid *D. sophia* at both the transcriptional and enzymatic activity levels.

### Key functional mutations in enzymatic activity between orthologous *UGT*s

Only three amino acids differed between Dsir06G01579 and Dscd6AG01520 (Dsir06G01579 A31, A64, and F213; Dscd6AG01520 V31, P64, and S213) (Supplementary Fig. S7). However, the enzymatic activity of Dscd6AG01520 was higher than that of Dsir06G01579. To characterize the critical amino acids affecting enzymatic activity, six mutants (Dsir06G01579 A31V, Dsir06G01579 A64P, and Dsir06G01579 F213S; Dscd6AG01520 V31A, Dscd6AG01520 P64A, and Dscd6AG01520 S213F) were generated through reciprocal amino acid substitutions between Dsir06G01579 and Dscd6AG01520. These six purified recombinant proteins were employed in an *in vitro* enzyme assay. The enzymatic activities of Dscd6AG01520 S213F, Dsir06G01579 A31V, and Dsir06G01579 A64P were similar to that of Dsir06G01579, and the enzymatic activities of Dsir06G01579 F213S, Dscd6AG01520 V31A, and Dscd6AG01520 P64A were similar to that of Dscd6AG01520 when quercetin and Q3G were employed as the substrates, respectively (Fig. 4c–f and Supplementary Table S5). Using Q7G as the substrate, all point mutants of Dsir06G01579 (Dsir06G01579 A31V, Dsir06G01579 A64P, and Dsir06G01579 F213S) displayed dramatically increased activity compared to Dsir06G01579, suggesting that each of these three substitutions (A31V, A64P, and F213S) significantly increased the enzymatic activity of Dsir06G01579 when using Q7G as the substrate (Fig. 4g and h, and Supplementary Table S5). The activities of Dscd6AG01520 V31A and Dscd6AG01520 S213F declined slightly, while Dscd6AG01520 P64A exhibited similar activity to that of Dscd6AG01520, suggesting that none of these substitutions dramatically affected the enzymatic activity of Dscd6AG01520 on the Q7G substrate (Fig. 4g and h, and Supplementary Table S5). Together, these data indicated that F213 significantly decreased the enzymatic activity of Dsir06G01579, while S213 boosted the enzymatic activity of Dscd6AG01520 with quercetin and Q3G as substrates. All three corresponding substitutions significantly increased the enzymatic activity of Dsir06G01579 but had a weak effect on the enzymatic activity of Dscd6AG01520 with Q7G as the substrate. Because the 213th amino acid is a critical position for the enzymatic activity of Dsir06G01579 and Dscd6AG01520, structural modeling and molecular docking were performed. The results showed that the 213th amino acid was located outside the catalytic center and was not involved in substrate and sugar donor binding (Supplementary Fig. S8a and b). Together, these results indicate that the 213th amino acid plays an important role in the enzymatic activities of Dsir06G01579 and Dscd6AG01520, but its mechanism of action is distinct from directly modulating the catalytic center or the substrate and sugar-donor binding sites.

### Sugar donor specificity and promiscuity of functional *UGT*s

As Dscd6AG01520 exhibited the highest catalytic efficiency among the four UGTs of interest, further investigation was conducted to delineate the sugar donor specificity and promiscuity of this enzyme. The sugar donor specificity of Dscd6AG01520 was characterized utilizing quercetin as the substrate. The investigation encompassed the examination of seven potential sugar donors: UDP-glucose, UDP-glucuronic acid, UDP-arabinose, UDP-xylose, UDP-rhamnose, UDP-galactose, and ADP-glucose. *In vitro* enzyme assays revealed that UDP-xylose and ADP-glucose were the utilizable sugar donors, apart from the previously mentioned UDP-glucose (Fig. 5b and Supplementary Fig. S9). To further characterize the promiscuity of Dscd6AG01520, nine typical flavonoid aglycones, which share a high degree of structural resemblance to quercetin or Q3G, were tested as potential substrates, while UDP-glucose served as the sugar donor. These nine flavonoid aglycones include quercetin, Q3G, Q7G, Q3,7G, apigenin, kaempferol, naringenin, quercetin-3-glucuronide, and dihydromyricetin. *In vitro* enzyme assays showed that kaempferol could be catalyzed by Dscd6AG01520, in addition to quercetin, Q3G, and Q7G (Fig. 5c). We determined the optimal pH for Dscd6AG01520 by measuring its activity in HEPES buffer across a range of seven values (6.8, 7.0, 7.6, 8.0, 8.5, 9.0, and 9.8). The results suggested that low pH (pH 6.8 and pH 7.0) dramatically reduced the enzymatic activity of Dscd6AG01520, while high pH (pH 8.0, pH 8.5, pH 9.0, and pH 9.8) had a very weak negative effect on the enzymatic activity of Dscd6AG01520 (Supplementary Fig. S9). Furthermore, we also found that the presence or absence of Mn^2+^ had no effect on the enzymatic activity of Dscd6AG01520 (Supplementary Fig. S9).

## Discussion

In recent years, rapid advances have been made in the field of genomics [28]. Whole-genome sequencing, high-quality assembly, and plant genome annotation have contributed significantly to research on evolution, genomes, and gene function, especially the discovery of biosynthesis pathways for secondary metabolites in medicinal plants, as exemplified by literature on camptothecin, wogonin, tanshinones, and leonurine in *Camptotheca acuminata*, *Scutellaria baicalensis*, sage, and *Leonurus japonicus*/*Leonurus sibiricus*, respectively [29–32]. In the present study, we assembled the T2T reference genomes for both diploid and tetraploid *D. sophia*. Based on these reference genomes, we discovered that the ancestral genome of *D. sophia* had an ACK origin and that tetraploid *D. sophia* was an autotetraploid without subgenome dominance. In combination with correlation analysis between the *UGT* gene expression levels and quercetin glucoside contents in *D. sophia* seeds, this study identified critical candidate UGTs (*Dscd6AG01520* and its homoeologs) contributing to the high quercetin glucoside accumulation (Q3G and Q3,7G) in tetraploid *D. sophia*. Further *in vitro* enzyme assays, gene expression level analysis, and collinearity analysis showed that gene duplication and functional/expression evolution of the homoeologs of *Dscd6AG01520* led to high quercetin glucoside accumulation (Q3G and Q3,7G) in tetraploid *D. sophia* (Fig. 5d). These findings demonstrate that high-quality genome assembly and annotation combined with multiomics analysis provide a vital foundation for unraveling complex biosynthetic processes in medicinal plants.

Flavonoids are a large class of secondary metabolites [33], with more than 9000 flavonoids identified in plants to date [34]. Plant flavonoids are widely utilized in daily life for food and medicinal purposes. For example, many foods and wines contain important edible pigments, anthocyanins, and proanthocyanidins, which also function as taste-regulating components [34–36]. Plant flavonoids are employed as pharmaceutical agents, contributing to the prevention of osteoporosis, cardiovascular disease, and cancer [34, 36, 37]. This study found that flavonoids comprised the main secondary metabolites in *D. sophia* seeds, and genome duplication led to elevated flavonoid content in tetraploid *D. sophia* (Fig. 1c and d). Considering that *D. sophia* has a short life span, high seed yield, and an established transformation and gene-editing system [38], this species offers a promising tool for the study of flavonoid biosynthesis and regulation pathways and their evolution through genome duplication.

It has been reported that induced autopolyploidy holds potential in enhancing plant secondary metabolite biosynthesis, which is of great significance for boosting the production of secondary metabolites with pharmaceutical value in medicinal plants [39–43]. For instance, two research groups have reported that induced tetraploid *Catharanthus roseus* exhibited increases of 2- to 3-fold in terpenoid indole alkaloids and 2-fold in vincristine compared to diploid *C. roseus* [44, 45]. It has also been reported that induced tetraploid *Cichorium intybus* displayed a 1.9-fold increase in total phenolics and a 10-fold increase in chlorogenic acid [46]. However, the application of chemical inducers to cause autopolyploidy often results in many unfavorable outcomes, such as infertility and genetic instability. In contrast, natural autopolyploids exhibit the benefits of autopolyploidy and avoid the shortcomings of chemical inducers. In this study, we revealed that the quercetin glucoside content of tetraploid *D. sophia* seeds was much higher than that of diploid *D. sophia* seeds (more than 60-fold). Therefore, the discovery of natural autopolyploids can be used to screen superior medicinal plant species with high biomass production and phytopharmaceuticals. In addition to the significant increase in secondary metabolites, polyploid plants usually display enhanced plant vigor, productivity, and tolerance to biotic and abiotic stress. In future research, tetraploid *D. sophia* should be assessed to determine whether it exhibits these advantages.

Synthetic biology provides a sustainable and efficient approach to producing phytopharmaceuticals of great economic value [47, 48]. Elucidating the key biosynthetic processes of secondary metabolites is a prerequisite for this approach [47]. Recently, Jiang *et al.* characterized an important bifunctional cytochrome P450 enzyme, TOT1 (taxane oxetanase 1), which is involved in the biosynthesis of baccatin III, an anti-cancer drug. They further successfully produced baccatin III in tobacco by artificially reconstituting the entire biosynthetic pathway [49]. Another example is QS-21, which is a potent vaccine adjuvant and a key component of human vaccines for a wide range of serious diseases. After the entire biosynthetic pathway of QS-21 was identified, complete QS-21 biosynthesis was achieved in engineered yeast and tobacco [50, 51]. In the present study, highly efficient UGTs were characterized that could be critical components for quercetin glucoside synthesis in a microbial chassis. Additionally, given its established transformation and gene-editing system and flavonoid-rich characteristics [38] (Fig. 1c), *D. sophia* might serve as a plant for studying the regulation and biosynthesis of flavonoids.

UGTs belong to the plant family 1 glycosyltransferases. They contain a variable N-terminal region, which is involved in substrate recognition and binding, and the conserved C-terminal PSPG motif. The conserved C-terminal PSPG motif spans 44 amino acids and contributes to the interaction with the sugar donor. Experimental evidence has shown that the 1st (W), 4th (Q), 19th (H), 24th (S), and 27th (E) positions of the PSPG motif are critical for the enzymatic activity of UGTs [52]. Moreover, the mutations in two conserved residues (H and D) in the N-terminal region, which form a substrate–H–D triad, also lead to the loss of enzymatic activity [53]. In this study, the 213th amino acid of Dsir06G01579/Dscd6AG01520 was found to be an important position for enzymatic activity, while structural modeling analysis suggested that the 213th amino acid was located outside the catalytic center and was not involved in substrate and sugar donor binding (Supplementary Fig. S8a and b). Therefore, the mechanism of action needs to be further investigated.

In summary, this study reveals the mechanism underlying the high accumulation of quercetin glucosides in tetraploid *D. sophia*. This research demonstrates that multiomics analysis holds broad potential for elucidating the biosynthesis pathways of secondary metabolites of great phytopharmaceutical value. Additionally, *D. sophia* belongs to the Brassicaceae family, which includes *A. thaliana*, a widely utilized model plant. *D. sophia* is very closely related to *A. thaliana* (Fig. 3a), and the two species share many advantageous characteristics, including self-fertilization, a short life span, high seed yield, and a small genome. The high-quality T2T reference genome of *D. sophia* assembled in this study, together with our recently established *D. sophia* transformation and gene-editing system, may serve as the cornerstone for developing *D. sophia* into an emerging model medicinal plant [38]. We believe that genomic resources and fundamental studies on *D. sophia* can significantly promote fundamental research on medicinal plants, offering new insights into the therapeutic potential of natural botanical resources.

## Materials and methods

### Plant material and growth conditions

*D. sophia* plants were grown in a growth chamber under long-day conditions (16 h light/8 h dark) at 26°C. Seeds of *D. sophia* were sterilized with 10% (v/v) commercial bleach, sown on half-strength Murashige and Skoog (MS) medium in Petri dishes, then the Petri dishes were placed in growth chambers for germination.

### Metabolomics analysis

A 100-mg aliquot of *D. sophia* seed powder was extracted with 1.5 ml of extraction solvent (methanol/water = 80:20, v/v). The mixture was vortexed for 1 min, ultrasonicated for 30 min, and then centrifuged at 14 000 rpm and 4°C for 15 min. The supernatant was collected into a fresh tube and transferred to amber LC vials for LC–ESI–MS/MS analysis. Quality contro samples (QC) were generated by mixing 100 μl aliquots of each sample.

The structures of the metabolites were characterized by matching retention times, accurate molecular weights, secondary fragmentation spectra, and collision energies with local standard databases and public databases, including mzVault, mzCloud, and ChemSpider. The identification and annotation of metabolites were conducted with Compound Discoverer 3.0. The identified metabolites were analyzed using MetaboAnalyst (https://www.metaboanalyst.ca/).

### Qualitative metabolite detection

Aliquots of 100 mg of finely ground *D. sophia* seed powder were extracted with 1 ml of the 50% methanolic solution. After filtration through a 0.22-μm filter, the extraction was analyzed via LC–MS analysis as reported [54].

### Karyotype analysis

Root tips of *D. sophia* were treated with nitrous oxide for 2 h, then fixed with 90% acetic acid for 10 min. After washing with double-distilled water, the root tips were enzymatically digested in a solution containing 1% pectolyase and 2% cellulase (Yakult Pharmaceutical) at 37°C for 1 h. Metaphase chromosome preparations were then made following the method of Kato *et al.* [55]. Metaphase cells were stained with 4′,6-diamidino-2-phenylindole (Vector Laboratories, CA, USA). More than three metaphase plates per sample were analyzed. Images were captured with a Leica DM2500 fluorescence microscope (Leica, Wetzlar, Germany).

### Genome assembly and annotation

The genomes of *D. sophia* were assembled from HiFi long reads using hifiasm v0.19.6 with default parameters [56], followed by haplotig purging with Purge Haplotigs v1.1.3 to remove sequences with aberrant coverage [57]. Hi-C scaffolding was performed using the YaHS v1.2a.1 pipeline and Juicer v1.6 with default parameters [58], with manual adjustments and error correction conducted in Juicebox v2.20.00 to generate the chromosome-level assembly and visualize the Hi-C interaction heatmap. Telomeric and centromeric regions were identified using quartet v1.1.3 using default parameters [59]. Assembly quality was assessed with BUSCO v5.4.3 and Merqury v1.3 [60, 61], while TEs were annotated using the EDTA v2.1.0 pipeline with sensitive 1 mode and additional repeat libraries (*A. thaliana* TE dataset) [62]. Gene models were predicted through the Maker v3.01.04 pipeline, integrating *ab initio* predictions with BRAKER v3.0.6 (trained with RNA-seq data), homolog proteins (*A. thaliana*), and RNA-seq-based transcript evidence (Supplementary Table S7) [63]. Functional annotation was carried out with eggNOG-mapper v2.1.10 [64, 65].

### Identification of syntenic genes and genomic fragments

Syntenic orthologs were identified among diploid and tetraploid genomes of *D. sophia* and 14 other Brassicaceae species, including *A. thaliana*, *A. lyrata*, *Aethionema arabicum*, *Arabis alpina*, *Brassica rapa*, *Brassica oleracea*, *C. rubella*, *Isatis indigotica*, *Leavenworthia alabamica*, *Raphanus sativus*, *Sisymbrium irio*, *Schrenkiella parvula*, *Thellungiella halophila*, and *C. papaya*, using SynOrths [66]. *A. thaliana* served as the reference genome, with others designed as queries. Coding sequences of syntenic orthologs were aligned with MUSCLE [67], and a neighbor-joining phylogenetic tree was generated from concatenated alignment using MEGA [68]. To estimate divergence times, the phylogenetic tree was time calibrated using MCMCTree with calibration points based on TIMETREE5 (http://timetree.org/). Four dated ages were chosen as calibration constraints, *A. lyrata* and *A. thaliana* divergence (5.09–10.41 Mya); *C. rubella* and *Arabidopsis* lineage split (8.03–15.84 Mya); Brassiceae tribe diversification including *R. sativus*, *B. oleracea*, *B. rapa*, and *S. irio* (9.82–24.63 Mya); and the root calibration representing early Brassicaceae divergence (70.5–89.6 Mya). For paralogous gene pairs in tetraploid *D. sophia*, MUSCLE-aligned sequences were analyzed with KaKs calculator to estimate synonymous substitution rates (Ks) [67, 69].

Based on the syntenic gene pairs identified between *D. sophia* and *Arabidopsis*, large-scale syntenic genomic fragments were identified by connecting adjacent syntenic gene pairs. Due to factors of local structural variations and potential genome assembly errors in the *D. sophia* and/or *Arabidopsis* genome, local syntenic gene pairs may not be distributed immediately adjacent to other syntenic genes in one or both genomes. Thus, if the two pairs of syntenic genes were separated by fewer than 50 intervening genes or within 300 kb genomic distance, they were consolidated into one pair of syntenic fragments. These identified syntenic fragments between *D. sophia* and *Arabidopsis* were mapped to the ACK system, based on the genomic associations in the ACK.

### Deciphering the ancestral diploid genome of *D. sophia*

Syntenic gene pairs that were consistently distributed across the *D. sophia* genome and ACK genomic blocks (GBs) were identified as ancestral genomic fragments inherited from the progenitor species. These identified syntenic fragments shared between ACK and *D. sophia* genomes were used to map ACK-derived GB information to *D. sophia* genomes. Each ACK GB corresponded to one copy in the diploid *D. sophia* and two copies in the tetraploid genome. We further examined GB associations in *D. sophia* and compared them with those in ACK. If the two subgenomes of tetraploid *D. sophia* shared identical GB associations, we considered that they originated from the ancestral diploid genome of *D. sophia*.

### Subgenome dominance analysis

The ratio of retained genes between the two subgenomes was analyzed using ACK genome as a reference. The chromosomes of tetraploid *D. sophia* were classified into two subgenomes based on gene density [70]. In detail, the set of chromosomes with higher gene density was designated as subgenome A (DsA), while the remaining chromosomes were grouped as subgenome B (DsB). The expression levels between paralogous gene pairs from the two subgenomes were compared using the mRNA-seq data from tetraploid *D. sophia* (Supplementary Table S7). Paralogous gene pairs with expression differences greater than 1- to 8-fold were counted.

### RNA-Seq analysis and screening the candidate *DsUGT* genes

Raw sequencing reads were processed using Trim-Galore (https://github.com/FelixKrueger/TrimGalore) to remove adapter sequences and low-quality reads. Cleaned reads were mapped to CD37001 genome using HISAT2 [71]. StringTie pipeline was employed to quantify the transcript abundance in transcripts per million [72]. To identify *DsUGT* genes in *D. sophia*, the UDPGT domain (PF00201) HMM profile was retrieved from InterPro (https://www.ebi.ac.uk/interpro/). The protein sequences of IR1-002 and CD37001 were scanned using HMMER 3.4 (https://github.com/EddyRivasLab/hmmer). Additionally, a BLASTP search was performed against the goodUGTs protein sequences of *D. bourgaeana*, a relative species of *D. sophia* (https://pugtdb.biodesign.ac.cn/), with an *E*-value threshold of 1e-05 [73]. All candidate proteins were further validated using the Conserved Domain Database to confirm the presence of a complete cl10013 domain [74]. To identify the candidate *UGT*s potentially involved in quercetin glucoside biosynthesis, the Pearson correlation coefficients between *UGT* gene expression levels and the total content of quercetin glucosides (Q3G + Q7G) were calculated. Genes with a strong positive correlation (*r* > 0.75, *P <* 1e-05) were selected as candidate *UGT*s.

### Molecular cloning

Full-length *DsUGT* genes (*Dsir06G01579*, *Dscd6AG01520*, *Dscd6BG01552*, and *Dscd6BG01553*) were PCR amplified from *D. sophia* seeds using Platinum SuperFi II DNA Polymerase (Thermo Fisher Scientific, USA). Diploid *D. sophia* (IR1-002) served as the template for *Dsir06G01579*, while tetraploid *D. sophia* (CD37001) served as the template for *Dscd6AG01520*, *Dscd6BG01552*, and *Dscd6BG01553.* All amplified *DsUGT* genes were cloned into pMAL-c5X plasmid (New England Biolabs, USA). The point mutations (Dscd6AG01520S213F, Dscd6AG01520A31V, Dscd6AG01520P64A, Dsir06G01579V31A, Dsir06G01579A64P and Dsir06G01579F213S) were generated via site-directed mutagenesis. All primer sequences are provided in Supplementary Table S6.

### Recombinant protein expression and purification

The recombinant plasmids were transformed into *E. coli* Rosetta (DE3) competent cells (AngYuBio, China). Transformed cells were cultured in LB medium with 50 μg/ml carbenicillin at 37°C with shaking (200 rpm) until OD_600_ reached 0.6–0.8. Protein expression was induced with 0.1 mM isopropyl β-d-1-thiogalactopyranoside at 16°C for 15 h. Cells were harvested by centrifugation (4000 × *g* for 10 min at 4°C), resuspended in the 20 ml Column buffer (20 mM Tris–HCl, pH 7.4, 200 mM NaCl and 10 mM β-mercaptoethanol) and lysed by sonication. The lysate was centrifuged (9000 × *g*, 30 min, 4°C), and the supernatant was subjected to affinity purification using amylose resin (New England Biolabs). Protein concentration was determined with the Omni-Easy™ BCA assay kit (Epizyme Biotech).

### Enzyme activity assay

The reaction mixture consisted of 30 mM HEPES (pH 7.6), 500 μM UDP-glucose, 200 μM substrates (quercetin, Q3G, and Q7G), 5 mM MnSO_4_, 1 mM DTT, and 5 μg of recombinant proteins. The reactions were carried out at 30°C for 3 h. To characterize the catalytic promiscuity and sugar donor specificity of Dscd6AG01520, nine substrates (including quercetin, Q3G, Q7G, Q3,7G, quercetin-3-*O*-glucuronide, kaempferol, apigenin, naringenin, and dihydromyricetin) and seven sugar donors (including UDP-glucose, UDP-xylose, UDP-arabinose, UDP-galactose, UDP-glucuronic acid, UDP-rhamnose, and ADP-glucose) were tested. The optimal pH value for Dscd6AG01520 was determined by testing its activity in HEPES buffer at seven pH values (6.8, 7.0, 7.6, 8.0, 8.5, 9.0, and 9.8). After termination with methanol, the samples were centrifuged. The supernatant was filtered through a 0.22-μm membrane. High-performance liquid chromatography (HPLC) was performed on a C18 reverse-phase column (Agilent, USA) using a gradient elution program with 0.1% formic acid in water and methanol as mobile phases, with detection at 254 nm. The conversion rates were calculated based on HPLC peak areas, and kinetic parameters were determined by Michaelis–Menten equation fitting. All experiments were performed with three independent biological replicates. Data were presented as mean ± standard deviation (SD), with statistical analysis performed using ANOVA in GraphPad Prism 8.

### Reverse transcription polymerase chain reaction

Total RNA was extracted using the TRIzol method (Invitrogen, USA). First-strand cDNA was synthesized from 1 mg of RNA using a RevertAid First Strand cDNA Synthesis Kit (Thermo Fisher, USA). RT-qPCR was performed to quantify the relative expression levels of target genes using primers listed in Supplemental Table S6. *DsUBC21* was used as the reference gene [27]. The relative expression of genes was calculated using the ∆Ct method.

### Protein-ligand docking

The 2D/3D structures of quercetin and UDP-glucose were obtained from the PubChem database. The structure of Dscd6AG01520 was predicted using a local AlphaFold2 server [75]. Protein-ligand docking was performed using AutoDock Vina with a multiple-ligand docking protocol to analyze the interactions with Dscd6AG01520 and quercetin/UDP-glucose [76].

## Supplementary Material

Web_Material_uhaf335

## Data Availability

All the raw sequencing data generated for this project have been deposited at the Genome Sequence Archive (https://ngdc.cncb.ac.cn/gsa/) under BioProject accession No. PRJCA034923.
