# Supplementary figures and images for: Telomere-to-telomere genome assembly and multiomics analyses illustrate the high accumulation of quercetin glucosides in tetraploid Descurainia sophia
Source: Hortic Res. 2025 Dec 3;13(3):uhaf335. doi: 10.1093/hr/uhaf335 (PMC12966014; doi:10.1093/hr/uhaf335)

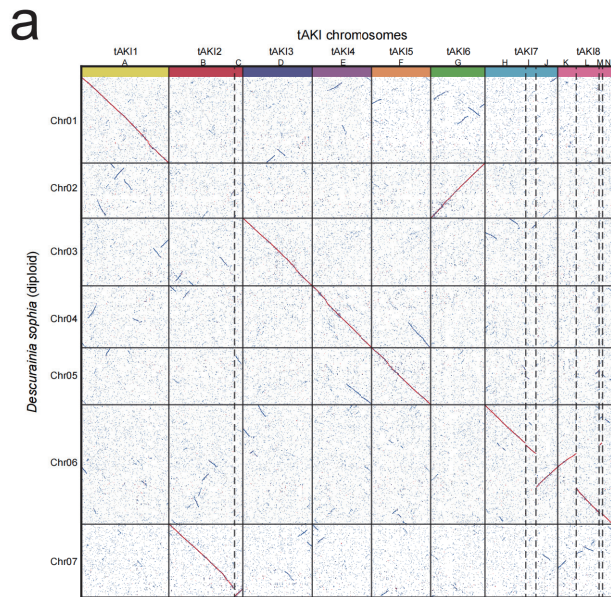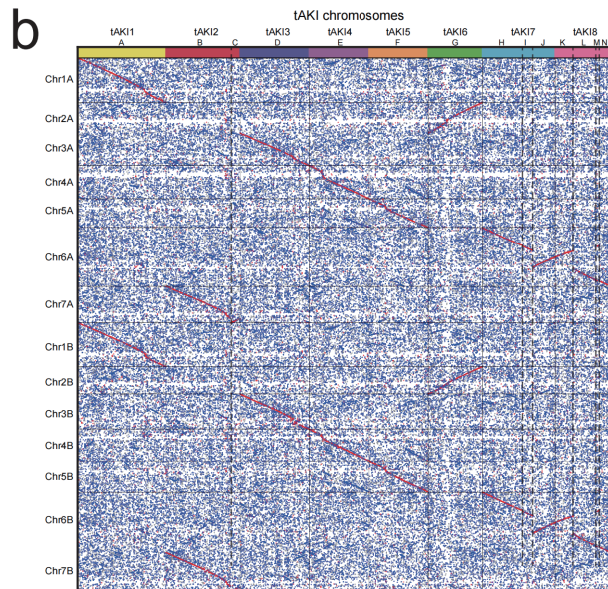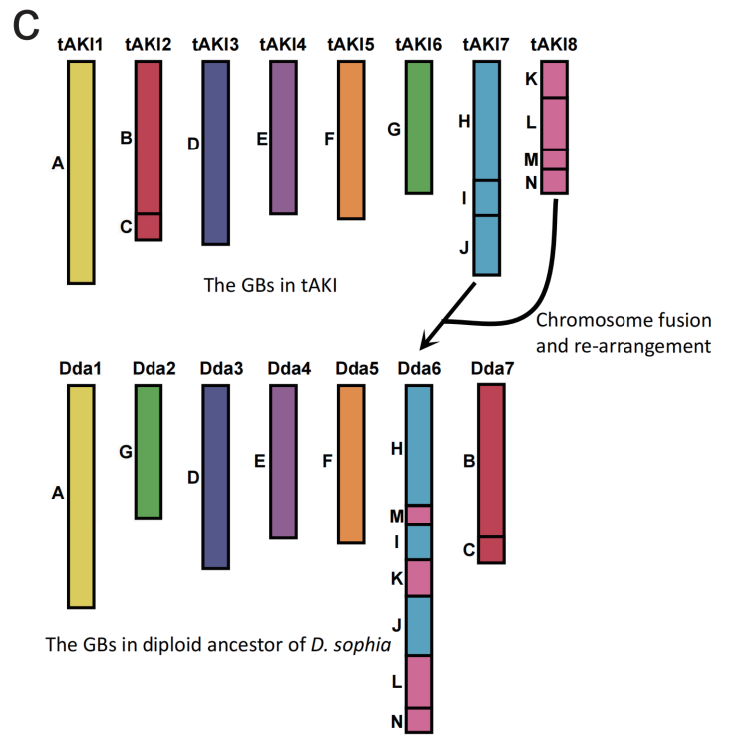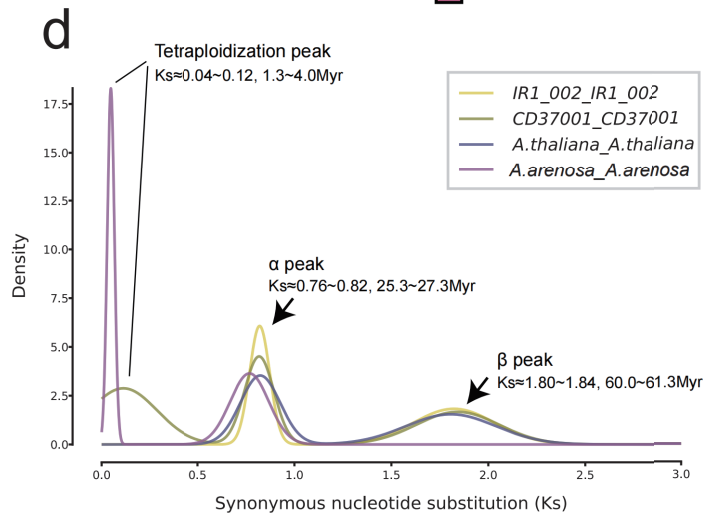

Supplement: Web_Material_uhaf335 [file web_material_uhaf335.zip › Supplementary Figure 3.pdf]

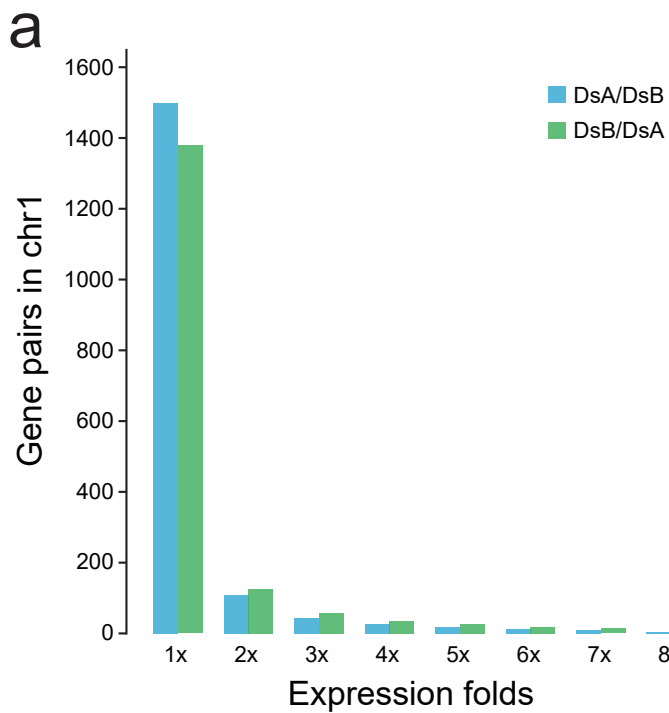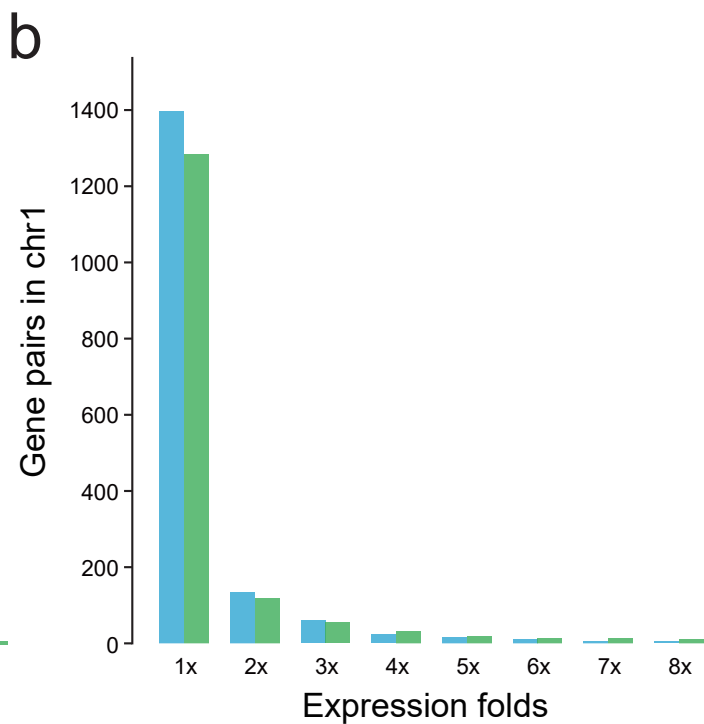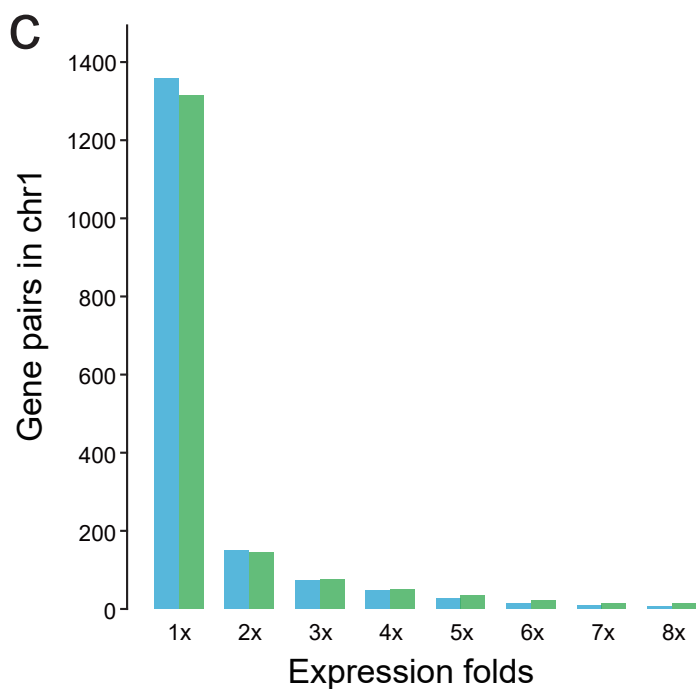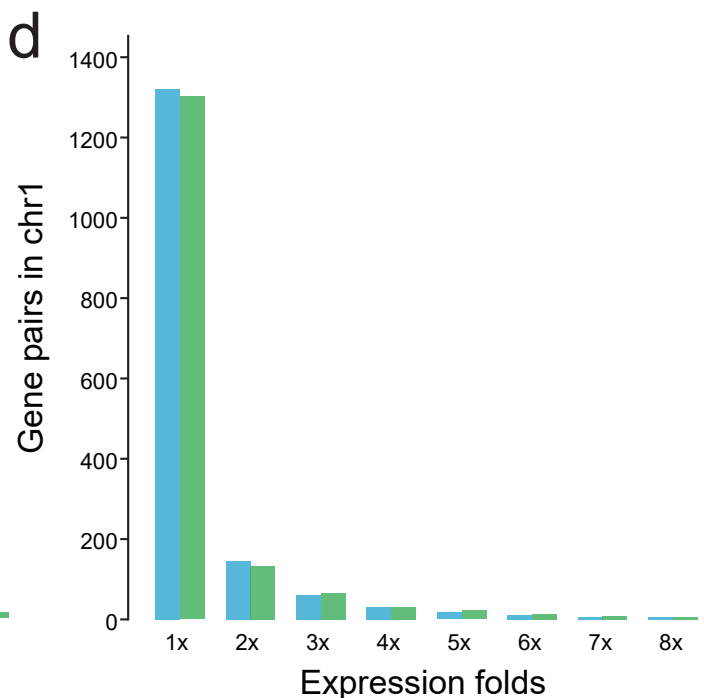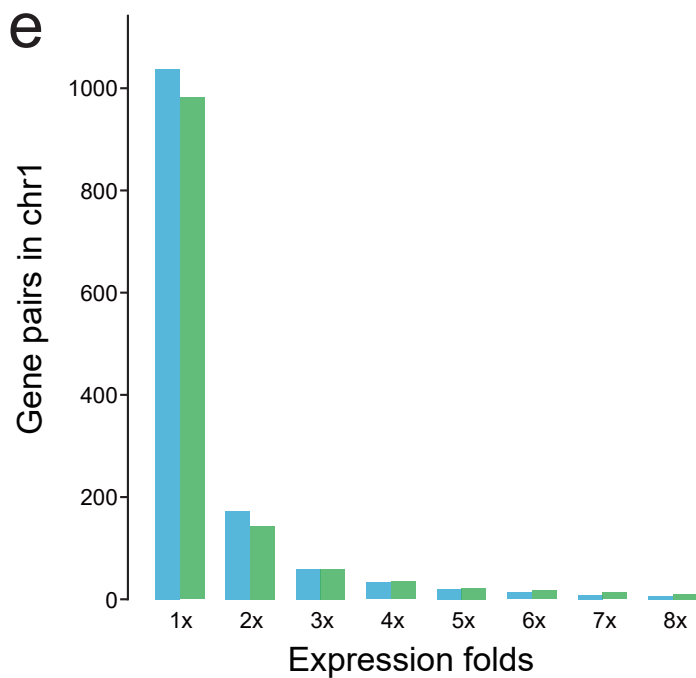

Supplement: Web_Material_uhaf335 [file web_material_uhaf335.zip › Supplementary Figure 4.pdf]

# IR1\_002

CD37001

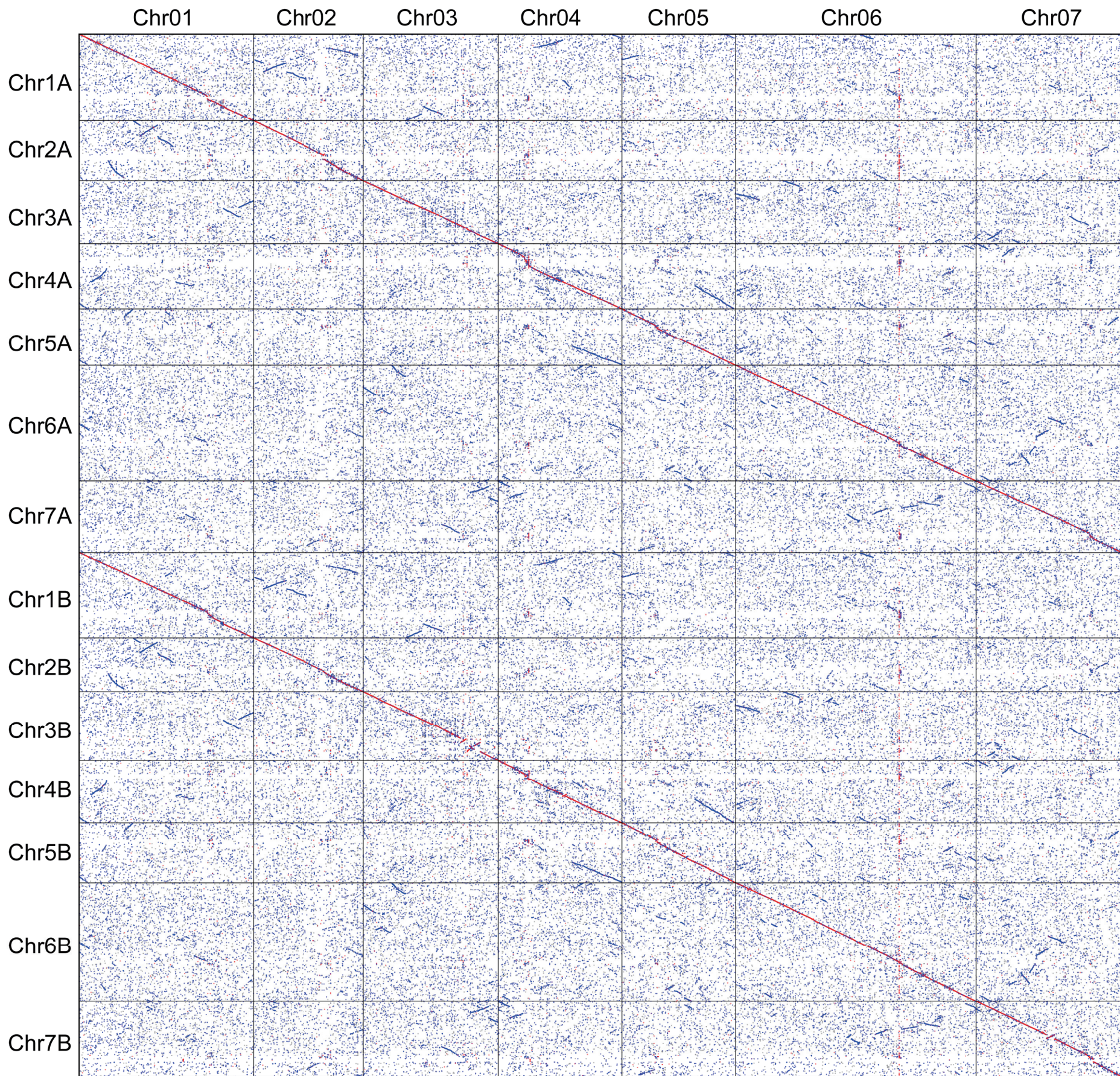

Supplement: Web_Material_uhaf335 [file web_material_uhaf335.zip › Supplementary Figure 5.pdf]
